# Retroviral activation of the mir-106a microRNA cistron in T lymphoma

**DOI:** 10.1186/1742-4690-4-5

**Published:** 2007-01-25

**Authors:** Amy M Lum, Bruce B Wang, Lauri Li, Namitha Channa, Gabor Bartha, Matthias Wabl

**Affiliations:** 1 Picobella, L.L.C., 863 Mitten Road, Suite 101, Burlingame, CA, 94010, USA; 2 Synergenics, L.L.C., 863 Mitten Road, Suite 101, Burlingame, CA, 94010, USA; 3 Department of Microbiology and Immunology, University of California, San Francisco, CA, 94143-0414, USA

## Abstract

Retroviral insertion into a host genome is a powerful tool not only for the discovery of cancer genes, but also for the discovery of potential oncogenic noncoding RNAs. In a large-scale mouse T lymphocyte tumor screen we found a high density of integrations upstream of the mir-106a microRNA cistron. In tumors containing an integration, the primary transcript encoding the mir-106a cistron was overexpressed five to 20-fold compared with that of control tumors; concomitantly, the mature mir-106a and mir-363 microRNAs were highly overexpressed as well. These findings suggest the mir-106a cistron plays an important role in T cell tumorigenesis.

## Findings

Retroviral insertions into the genome of a host can induce tumor formation by altering gene expression or function. Integration of a retrovirus near a gene can induce overexpression of the gene through the viral promoter or enhancer, while insertion of a retrovirus into a gene can cause both activation and inactivation. If the affected genes are proto-oncogenes or tumor suppressor genes, the insertion events may lead to tumor formation [1]. Consequently, retroviral mutagenesis has been used to search entire genomes for genes involved in cancer development [2-4], including oncogenic microRNAs (miRNAs) [5]. MiRNAs are short (~22 bp) noncoding RNAs that are implicated in gene regulation and cancer [6-10]. In a large-scale retroviral insertion mutagenesis screen, we used the murine leukemia virus (MLV) strain SL3-3, which causes T lymphomas [11], and identified several miRNAs that are potentially involved in tumorigenesis. We previously demonstrated that a group of these retroviral insertions induces overexpression of the oncogenic mmu-mir-17 miRNA cistron in mouse tumors [5]. Here we build on our validation of the retrovirus insertional mutagenesis method to identify oncogenic miRNA and present another potentially oncogenic miRNA cistron, mmu-mir-106a. In this screen, male BALB/c mice were treated with ethyl-nitroso-urea (ENU) and bred to normal female mice. ENU treatment was conducted to increase the recovery of tumor suppressors in the F1 progeny through mutagenesis of the paternal allele. Newborn offspring mice were then injected with MLV strain SL3-3. After becoming moribund due to tumor development, mice were euthanized and thymus and spleen tissues were collected and stored at -80°C. Locations of the SL3-3 provirus integration sites were identified as previously described using a splinkerette based PCR method [3] that amplifies genomic DNA flanking the 5' LTR of the virus.

**Figure 1:**
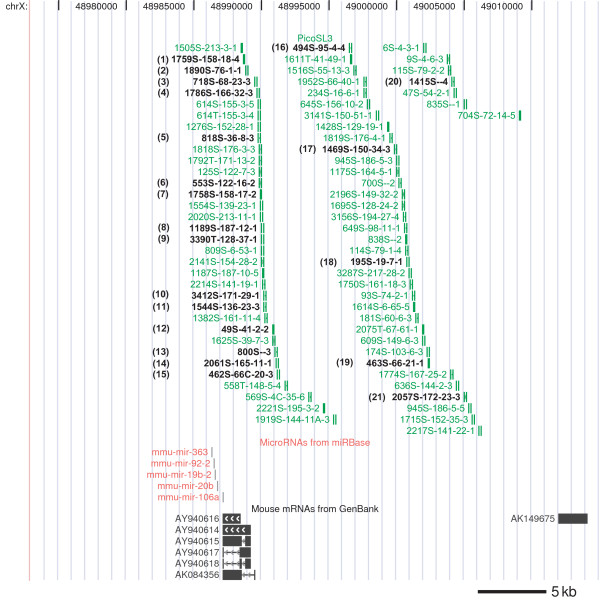
**Map of integration sites upstream of the mmu-mir-106a cistron**. A map of the SL3-3 retroviral sites upstream of the mir-106a cluster is displayed using the UCSC genome website browser (February 2006 version of the mm8 genome assembly). Insertion sites are depicted as vertical handlebars. Tumors assayed by quantitative PCR are numbered and noted in black text.

We identified 6234 integration sites in 2199 tumors; of these tumors, 76 sites were located on chromosome X upstream of a miRNA cluster containing mmu-mir-106a, mmu-mir-20b, mmu-mir-19b-2, mmu-mir-92-2, and mmu-mir-363. The locations of the integrations ranged from 1.5 kb to 22 kb upstream of the miRNA cluster (Fig. 1), with proviral inserts in both sense and anti-sense orientations with respect to the primary RNA transcript encoding the miRNA cistron. The Mouse Retroviral Tagged Cancer Gene Database [12], which compiles retroviral insertions into the genomic DNA from various non-T cell derived mouse tumors, also lists 10 integrations located upstream of the mmu-mir-106a cluster. Furthermore, Hwang et al. found that EST AI464896, which maps to the same location as mmu-mir-363, was overexpressed in tumors with proviral MLV integrations into this region [13]. The radiation leukemia virus (RadLV) also frequently integrates at this locus and a group of five differentially spliced noncoding RNAs known as Kis2 (GenBank Accession numbers AY940614-AY940618) are overexpressed in these tumors [14]. Because the Kis2 transcripts lie directly upstream of the mir-106a miRNA cluster (mmu-mir-106a overlaps these transcripts by four bases), they likely are part of the primary transcripts containing the miRNA cluster.

To determine whether the retroviral integrations in this region affected the expression of the mir-106a cistron, we used quantitative PCR (qPCR) to measure expression levels of the primary transcript (Kis2) and the mature miRNAs (mmu-mir-106a and mmu-mir-363) in tumors containing mir-106a cistron integrations as well as in control tumors lacking such integrations. To measure primary transcript (Kis2) expression levels, a probe and primer set was designed to AY940616, which is a common exon to three of the alternatively spliced forms of Kis2. The probe and primers for AY940616 were as follows: 5'-TGTGTCCCTGAAGTTTATTGGTGT-3', 5'-GGGTCACGAGCTCCCTCC-3', and 5'-[6-FAM]-CCCCCATCAACACAAACATTCCATCA-[3BHQ1]-3'. MiRNAs and low molecular weight RNAs were isolated from frozen mouse tumor tissue using the Purelink miRNA Isolation Kit (Invitrogen). Large fraction RNAs were then purified by eluting the high molecular weight RNA bound to the first column (used for the miRNA purification). cDNA was generated from total RNA by reverse transcription with random hexamers using the SuperScript First-Strand Synthesis System for RT-PCR (Invitrogen). qPCR runs were conducted on the MX3000P (Stratagene). All qPCR reactions were run in triplicate. As controls, tumors not containing integrations near the mmu-mir-106a-363 cluster were also assayed. Beta-actin was used as the endogenous reference gene (Mouse ACTB 20× VIC-MGB probe set, Applied Biosystems) and control tumor 1 was used as the calibrator sample in the calculation of 2^-ΔΔCt ^values (relative expression). All relative expression values were normalized such that the average of the tumor controls was set to 1.

**Figure 2:**
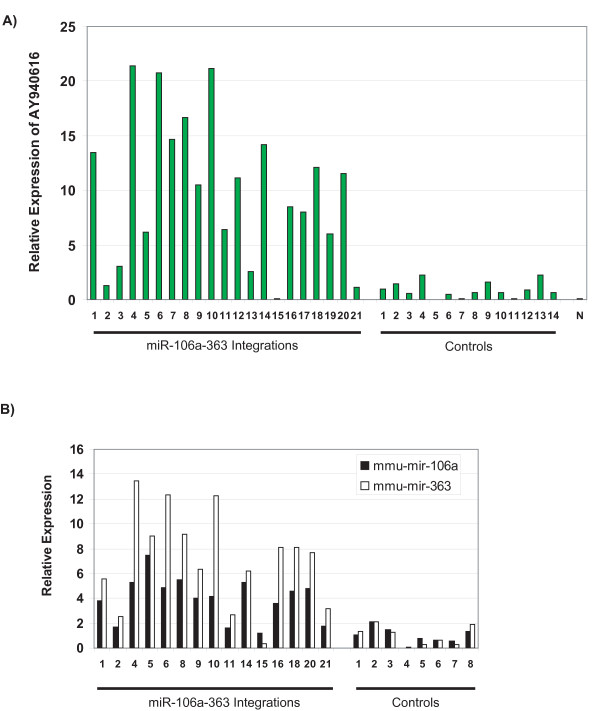
**Expression of the primary transcript and mature species of the mmu-mir-106a cistron**. Quantitative PCR data for tumors with integrations upstream of the mir-106a cistron. (A) Relative expression of AY940616 in tumors containing integration sites near the mir-106a miRNA cluster. Control tumors contain integration sites at locations in the genome other than the mir-106a region. "N" is cDNA generated from normal mouse spleen RNA (Ambion). (B) Relative expression of the mature species of mmu-mir-106a and mmu-mir-363 in tumors containing integration sites near the mir-106a cistron. Tumors are numbered as in Figure 1.

Representative tumors with integration sites spanning the upstream region of mir-106a were measured for expression of the miRNA primary transcript (Fig. 1 and Table 1). In 16 of the 21 tumors assayed, expression of AY940616 was elevated five to 20 fold as compared to the average expression of tumors with no integrations at this locus (Fig. 2A). This confirms the previous report that proviral integrations in this region can increase expression of the Kis2 locus [14].

**Table 1:** Assayed mmu-mir-106a cistron integrations

**#**	**Tumor**	**Location**	**Orientation**
1	1759S-158-18	chrX:48988832	G-T-
2	1890S-76-1	chrX:48988834	G-T+
3	718S-68-23	chrX:48989486	G-T+
4	1786S-166-32	chrX:48989857	G-T-
5	818S-36-8	chrX:48989770	G-T+
6	553S-122-16	chrX:48990068	G-T-
7	1758S-158-17	chrX:48989877	G-T+
8	1189S-187-12	chrX:48989980	G-T+
9	3390T-128-37	chrX:48990180	G-T-
10	3412S-171-29	chrX:48990192	G-T+
11	1544S-136-23	chrX:48990391	G-T-
12	49S-41-2	chrX:48991005	G-T-
13	800S-	chrX:48991024	G-T+
14	2061S-165-11	chrX:48991259	G-T-
15	462S-66C-20	chrX:48991159	G-T+
16	494S-95-4	chrX:48996521	G-T+
17	1469S-150-34	chrX:48999846	G-T+
18	195S-19-7	chrX:49000742	G-T+
19	463S-66-21	chrX:49002308	G-T+
20	1415S-	chrX:49004115	G-T+
21	2057S-172-23	chrX:49005225	G-T-

Retroviral insertion site locations (February 2006 version of the UCSC mm8 genome assembly) are noted by the basepair located just before the insertion. Orientation of the retrovirus is described using the following nomenclature. The gene transcript (G) and retroviral tag (T) is directly followed by a "+" for directionality of left to right or by a "-" for directionality of right to left on the chromosome. If the gene transcript is located to the left of the retroviral tag on the chromosome, the notation for the gene precedes the notation for the tag (G ± T±). If the transcript is to the right of the retroviral tag on the chromosome the order is reversed (T ± G±). If the tag is located within the gene, by default the gene precedes the tag annotation.

The mature species of mmu-mir-106a and mmu-mir-363 were then measured by RT-qPCR using a stem-loop RT primer specific for each miRNA [15]. Accordingly, 50 ng of each tumor miRNA preparation was reverse transcribed with the SuperScript First-Strand Synthesis System for RT-PCR using the following stem loop RT primers (50 nM final concentration) 5'-GTCGTATCCAGTGCAGGGTCCGAGGTATTCGCACTGGATACGACTACCTG-3'(mmu-mir-106a) and 5'-GTCGTATCCAGTGCAGGGTCCGAGGTATTCGCACTGGATACGACTTACAG-3' (mmu-mir-363). The reverse transcription reactions were diluted 1:200 and 5 μl of these dilutions were used in the 25 μl qPCR reactions. The annealing step was 50°C for 60s. The qPCR probes and primers were as follows: mmu-mir-106a: 5'-CGGCAAAGTGCTAACAGT-3', 5'-GTGCAGGGTCCGAGGT-3', 5'- [6-FAM]- CACTGGATACGACTACCTGC- [BHQ1]-3'; and mmu-mir-363: 5'-TGCGGATTGCACGGTATC-3', 5'-GTGCAGGGTCCGAGGT-3', 5'- [6-FAM]- CACTGGATACGACTTACAGATG- [BHQ1]-3'. Synthetic RNA oligos (IDT) were used to generate a calibration curve for each miRNA: 5'-CAAAGUGCUAACAGUGCAGGUA-3' (mmu-mir-106a) and 5'-AUUGCACGGUAUCCAUCUGUAA-3'(mmu-mir-363). Amplification efficiencies of the calibration curves for mmu-mir-106a and mmu-mir-363 were respectively 67% and 69%. Concentrations of the mature species were calculated using the calibration curves and then normalized by the average of the control tumors, to calculate relative expression levels.

Fifteen tumors with integrations in this region were assayed by qPCR for the mature species of mmu-mir-106a and mmu-mir-363. Approximately 70% of these tumors had increased expression levels of mmu-mir-106a by two to six fold, and of mmu-mir-363 by four to 12 fold over the average expression of tumors with no integrations in this region (Fig. 2B). The mature miRNA expression difference between tumors with integrations in this region and the tumor controls was statistically significant [p < 0.00001 (mmu-mir-106a) and p < 0.0001 (mmu-mir-363)] by a two sample unequal variance Student's t test. From these data we conclude that retroviral integrations in the Kis2 region cause overexpression not only of the primary RNA, but also of the mature species of the mir-106a cluster. This, in turn, suggests that the miRNA cluster can drive the development of T lymphomas. Although there is a possibility that these integrations also may affect the expression of other oncogenes and tumor suppressors in this region, our data clearly indicates a majority of these integrations induce the expression of the mir-106a cluster.

As the mir-106a cistron is a homolog of the oncogenic mir-17 cistron [16], it is not unexpected that mir-106a would also be involved tumorigenesis. Indeed, in human solid tumors, mir-106a expression is increased in colon, pancreas, and prostate tumors; and mir-92-2 expression is increased in pancreas, prostate, and stomach tumors [17]. Given the sequence similarity between the mir-17 and mir-106a cistrons, it is likely that these clusters have overlapping gene targets. In humans, the mir-106a cistron contains several paralogs to members of the mir-17 cistron including mir-17, mir-19b-1, and mir-92-1 [16], which are implicated in cancer development: overexpression of the mir-17 cluster accelerates lymphoma formation from cells of mice overexpressing c-Myc [7]. The mir-17 cluster is also overexpressed in human lung cancer [18]. However, in breast cancer cells, mir-17-5p expression is decreased; there it acts as a translational repressor of the oncogene AIB1 (amplified in breast cancer 1) [19], and in this context may formally act as a tumor suppressor.

It is well established that tumorigenesis is the result of accumulating several cooperating mutations that drive relentless proliferation and aid in metastases. Viral insertional mutagenesis, though perhaps not providing all the mutations necessary for a full-blown tumor, follows this multistep scenario. Although in general the superinfection barrier largely prevents multiple proviral integrations within the same cell, re-infection does happen over time. Because it is a rare event, such cells are selected over the others only when these integrations also give a growth advantage. As a consequence, in general, most viral insertions ("co-mutations") in a single tumor are thought to be causative in its formation. With the caveats of potential passenger genes and potential oligoclonality of tumors, co-mutation analysis may be a powerful way to find cooperating signaling pathways in tumorigenesis.

We detected multiple insertion sites in all of the tumor samples we assayed from the mir-106a cluster. Genes near common co-integration sites for these tumors include Ahi1, Evi5, and Gfi1, candidates previously appearing in retroviral screens [12], as well as PVT1, a noncoding RNA frequently amplified with myc [20]. A summary of all integration sites in the assayed tumors is listed in Table 2.

**Table 2:** Summary of integrations in tumors assayed for the mmu-mir-106a cistron

				***Gene located near insertion site***	
**Tumor #**	**Tumor Name**	**Location**	**Orientation**	**Abbr/Acc**	**Description**
1	1759S-158-18	chr18:78192824	G+T+	XM_973419	
1	1759S-158-18	chr7:73480330	T-G+	XM_978127.1	
1	1759S-158-18	chr5:107965444	G-T-	Gfi1	Growth factor independent 1
2	1890S-76-1	chr10:20756607	G+T-	Ahi1	Jouberin
2	1890S-76-1	chr7:144921997	T-G-	Tpcn2	Two pore segment channel 2
2	1890S-76-1	chr15:61868694	G+T+	PVT1	Plasmacytoma variant translocation 1
3	718S-68-23	chr17:29126185	G+T-	Fgd2	FYVE, RhoGEF and PH domain containing 2
3	718S-68-23	chr11:5819233	G-T-	Gck	Glucokinase
3	718S-68-23	chr11:66037536	G-T-	Gm879	Gene model 879
4	1786S-166-32	chr5:116571294	G-T-	Ccdc60	Coiled-coil domain containing 60
4	1786S-166-32	chr5:107977381	T+G-	Evi5	Ecotropic viral integration site 5
5	818S-36-8	chr18:5348771	G-T-	Zfp438	Zinc finger protein 438
5	818S-36-8	chr5:15380286	T-G+	Cacna2d1	Calcium channel, voltage-dependent, alpha2/delta subunit 1
6	553S-122-16	chr5:107957997	G-T-	Gfi1	Growth factor independent 1
6	553S-122-16	chr15:62006727	G+T-	PVT1	Plasmacytoma variant translocation 1
7	1758S-158-17	chr5:115421225	G-T-	2410014A08Rik	Hypothetical protein LOC109154
7	1758S-158-17	chr5:107968359	G-T+	Gfi1	Growth factor independent 1
7	1758S-158-17	chr17:46990647	G-T-	Tbn	Taube nuss
8	1189S-187-12	chr5:107970686	G-T+	Gfi1	Growth factor independent 1
8	1189S-187-12	chr9:20880975	G-T-	Tyk2	Tyrosine kinase 2
8	1189S-187-12	chr15:63293889	T+G-	XM_139402	
8	1189S-187-12	chr19:55328465	G+T-	Acsl5	Acyl-CoA synthetase long-chain family member 5
9	3390T-128-37	chr2:117124415	G-T+	Rasgrp1	RAS guanyl releasing protein 1
9	3390T-128-37	chr7:113933436	G-T+	Rras2	Related RAS viral (r-ras) oncogene homolog 2
9	3390T-128-37	chr7:113636412	G+T-	Spon1	Spondin 1, (f-spondin) extracellular matrix protein
9	3390T-128-37	chr10:20761837	G+T-	Ahi1	Jouberin
10	3412S-171-29	chr10:20781072	G+T-	Ahi1	Jouberin
11	1544S-136-23	chr13_random:67900	G+T+	NM_175538.2	RIKEN cDNA E130304F04 gene
11	1544S-136-23	chr9:36980695	G-T+	Slc37a2	Solute carrier family 37 (glycerol-3-phosphate transporter), member 2
11	1544S-136-23	chr10:58965185	G-T+	NM_001033259.1	RIKEN cDNA D130073L02 gene
12	49S-41-2	chr2:72016508	G+T+	Rapgef4	Rap guanine nucleotide exchange factor (GEF) 4
12	49S-41-2	chr17:47006160	G-T-	Tbn	Taube nuss
13	800S-	chr7:58627272	G+T-	Atp10a	ATPase, class V, type 10A
13	800S-	chr16:94677486	G-T-	Dscr3	Down syndrome critical region gene 3
14	2061S-165-11	chr14:77172712	T+G-	Akap11	A kinase (PRKA) anchor protein 11
14	2061S-165-11	chr5:107977456	T+G-	Evi5	Ecotropic viral integration site 5
14	2061S-165-11	chr17:29125847	G+T-	Fgd2	FYVE, RhoGEF and PH domain containing 2
15	462S-66C-20	chr2:26317278	G-T+	Notch1	Notch gene homolog 1 (Drosophila)
15	462S-66C-20	chr10:20779700	G+T-	Ahi1	Jouberin
15	462S-66C-20	chr12:86435291	G+T-	XM_988509	
16	494S-95-4	chr10:120923128	T+G-	Tbk1	TANK-binding kinase 1
16	494S-95-4	chr14:121149083	G+T+	Phgdhl1	Phosphoglycerate dehydrogenase like 1
16	494S-95-4	chr1:139964759	G-T+	Ptprc	Protein tyrosine phosphatase, receptor type, C
17	1469S-150-34	chr16:48708118	G+T+	LOC432823	
17	1469S-150-34	chr5:107979863	T+G-	Evi5	Ecotropic viral integration site 5
17	1469S-150-34	chr12:86550688	G+T+	Jundm2	Jun dimerization protein 2
18	195S-19-7	chr10:20744863	G+T-	Ahi1	Jouberin
19	463S-66-21	chr4:98038839	G+T-	Inadl	InaD-like (Drosophila)
19	463S-66-21	chr5:107968538	G-T+	Gfi1	Growth factor independent 1
19	463S-66-21	chr17:46998042	G-T-	Tbn	Taube nuss
19	463S-66-21	chr1:137623944	G+T-	Tnni1	Troponin I, skeletal, slow 1
20	1415S-	chr15:61998664	G+T+	PVT1	Plasmacytoma variant translocation 1
20	1415S-	chr14:68239584	G-T+	Slc25a37	Solute carrier family 25, member 37
20	1415S-	chr14:78271471	G+T-	Elf1	E74-like factor 1
21	2057S-172-23	chr15:79832811	G-T+	Pdgfb	platelet derived growth factor, B polypeptide
21	2057S-172-23	chr13:52820992	T-G-	Auh	AU RNA binding protein/enoyl-coenzyme A hydratase

Additional retroviral insertion sites recovered from tumors containing an insertion site upstream of the mir-106a cistron. Retroviral insertion site locations and orientations are notated as in Table 1. Nearby genes to the insertion sites are also listed.

Through retroviral insertion in the mouse, we have discovered another potentially oncogenic microRNA cluster, mir-106a-363. Retroviral insertion caused significant overexpression of this microRNA cluster indicating its role in tumor development. This study further demonstrates the power of retrovirus insertion as a tool to discover new oncogenic noncoding RNAs.

## Competing interests

The authors declare a financial interest in Picobella, LLC.

## Authors' contributions

AML carried out the RNA isolation, quantitative PCR, expression data analysis, and drafted the manuscript. GB, LL, NC, and BBW carried out the tag recovery and identification. BBW and MW planned and directed the execution of the retroviral screen, the design of the study, and the writing of the manuscript.
